# ANCA-associated vasculitis is associated with an increased risk of cardiac and vascular morbidity: results of a large-scale propensity-matched global retrospective cohort study

**DOI:** 10.3389/fimmu.2026.1794549

**Published:** 2026-04-15

**Authors:** Sebastian Klapa, Katja Bieber, Artem Vorobyev, Marlene Ludwig, Gordon Vollert, Sabrina Arnold, Henning Olbrich, Henry Nording, Antje Müller, Khalaf Kridin, Jan Henrik Schirmer, Katja von Allwörden, Anja Staehle, Ralf Ludwig, Peter Lamprecht

**Affiliations:** 1 Department of Rheumatology and Clinical Immunology, University of Lübeck, Lübeck, Germany; 2 Institute of Experimental Medicine, Christian-Albrechts-University of Kiel c/o German Naval Medical Institute, Kronshagen, Germany; 3 Lübeck Institute of Experimental Dermatology, University of Lübeck, Lübeck, Germany; 4 Independent Researcher, Groß Grönau, Germany; 5 Department of Dermatology, University of Lübeck, Lübeck, Germany; 6 DZHK (German Centre for Cardiovascular Research), Partner Site North, Lübeck/Kiel, Germany; 7 Department of Internal Medicine V, Angiology, Christian-Albrechts-University of Kiel, Kiel, Germany; 8 Azrieli Faculty of Medicine, Bar-Ilan University, Safed, Israel; 9 Clinic for Internal Medicine I, Rheumatology and Clinical Immunology, University Medical Center Schleswig-Holstein Campus Kiel, Kiel, Germany

**Keywords:** ANCA - associated vasculitis, cardiovascular outcome, granulomatosis with poliangiitis, microscopic polyangiitis, morbididy, mortality, MACE, thrombembolic events

## Abstract

**Introduction:**

Despite significantly improved therapies in recent years, long-term morbidity and mortality in ANCA-associated vasculitis (AAV) remain high. The leading causes of death within the first year after diagnosis are active vasculitis and in subsequent years cardiovascular diseases, malignancies, and infections. Population-based database and cohort analyses suggest an increased risk for major adverse cardiovascular events (MACE) in AAV.

**Methods:**

This retrospective cohort study analyzed data samples from an electronic health records database of the US-based TriNetX network. Patients with the diagnostic codes granulomatosis with polyangiitis (GPA) or microscopic polyangiitis (MPA) and patients without vasculitis as a matched control cohort (1:1) were included. To optimize between-group comparability, propensity score matching was performed for demographic variables and comorbidity. Hazard ratios (HR) for death and cardiovascular outcomes were calculated using univariate Cox regression after analyzing the matched cohort using the Kaplan-Meier method.

**Results:**

We identified 20, 422 patients with GPA and 5, 907 with MPA. Mortality was more frequent in patients with GPA (17.87%) and MPA (25.85%) than in matched controls (GPA controls: 5.79%; MPA controls: 9.70%), corresponding to an increased hazard of death in both cohorts (GPA: HR 3.01; MPA: HR 3.01). The risk of cardiovascular events was increased in GPA and MPA compared to matched controls, particularly for MACE (GPA: HR: 1.94, MPA: HR: 2.24) and thromboembolic events (deep vein thrombosis: GPA HR: 2.82, MPA HR: 3.33; pulmonary embolism: GPA HR: 3.01, MPA HR: 3.00) and did not differ when adjusted according to sex, disease duration, and age. Compared with GPA patients, MPA patients had a higher risk of MACE (HR: 1.13) and peripheral arterial disease (HR: 1.17).

**Conclusion:**

AAV was associated with an increased risk of death and cardiovascular events. Compared with GPA, MPA was associated with an increased risk for MACE and peripheral arterial disease.

## Introduction

Antineutrophil cytoplasmic antibody (ANCA)-associated vasculitis (AAV) is characterized by a systemic necrotizing vasculitis predominantly affecting small and medium-sized vessels. The two major subgroups of AAV are granulomatosis with polyangiitis (GPA) and microscopic polyangiitis (MPA) (1–3). Despite significant improvement in the efficacy of immunosuppressive treatments, GPA and MPA continue to be associated with significant morbidity and mortality risks (4). Prospective randomized controlled trials showed patients with AAV are at increased risk of death due to active vasculitis and infections in the first year after diagnosis and in subsequent years due to cardiovascular disease, malignancies, infections, and active vasculitis (4–6). Major adverse cardiovascular events (MACE) defined as acute cerebral and myocardial infarction, congestive heart failure (CHF), ventricular arrhythmia (VA) and sudden cardiac death, are a major contributor to mortality within one year of an AAV diagnosis (7–10). By contrast, a monocentric, population-based cohort study found no increased risk of cardiovascular death or MACE despite an increased risk of AF and CHF (11). However, there is a lack of real-world data on mortality and cardiovascular outcomes in AAV and comparison of outcomes between GPA and MPA.

In the present study, we conducted a large-scale, propensity-matched, global, retrospective cohort study using electronic health records (EHRs) provided by the Global Collaborative Network of TriNetX (12). The EHRs of 66 healthcare organisations (HCOs) contain daily data on diagnoses (ICD-10), medications, and medical procedures (12). We assessed the risks of mortality and cardiovascular diseases after diagnosis of GPA or MPA. We compared these risks within and between patient groups, with both groups matching for Framingham cardiovascular risk factors and other major comorbidities (13).

## Methods

### Study design, database and study population

A global propensity-score matched retrospective cohort study was conducted adhering to established procedures (14–16). In detail, electronic health records (EHRs) from patients with AAV were retrieved from the US Collaborative Network (with natural language processing) within the federated TriNetX platform (12). At baseline, this network provided access to 116, 685, 316 EHRs from 66 Health Care Organisations (HCOs) located in the USA and Europe (16). The following cohorts, described in detail below, were identified: (i) ANCA-vasculitis (ICD10CM:M31.3 or M31.7), (ii) granulomatosis with polyangiitis (M31.3), (iii) microscopic polyangiitis (M31.7), and (iv) non-systemic vasculitis controls - ICD10CM:Z00, encoding for “Encounter for general examination without complaint, suspected or reported diagnosis” without any diagnostic code for M30.0 (polyarteritis nodosa), M30.1, M31.3, M31.6, or M35.3. The index event was defined as diagnosis of AAV or healthcare encounter. Predefined outcomes were death and cardiovascular diseases. Propensity score matching (PSM) was used to enhance comparability among groups, and sensitivity analyses were included to strengthen the study’s robustness.

**Figure 1:**
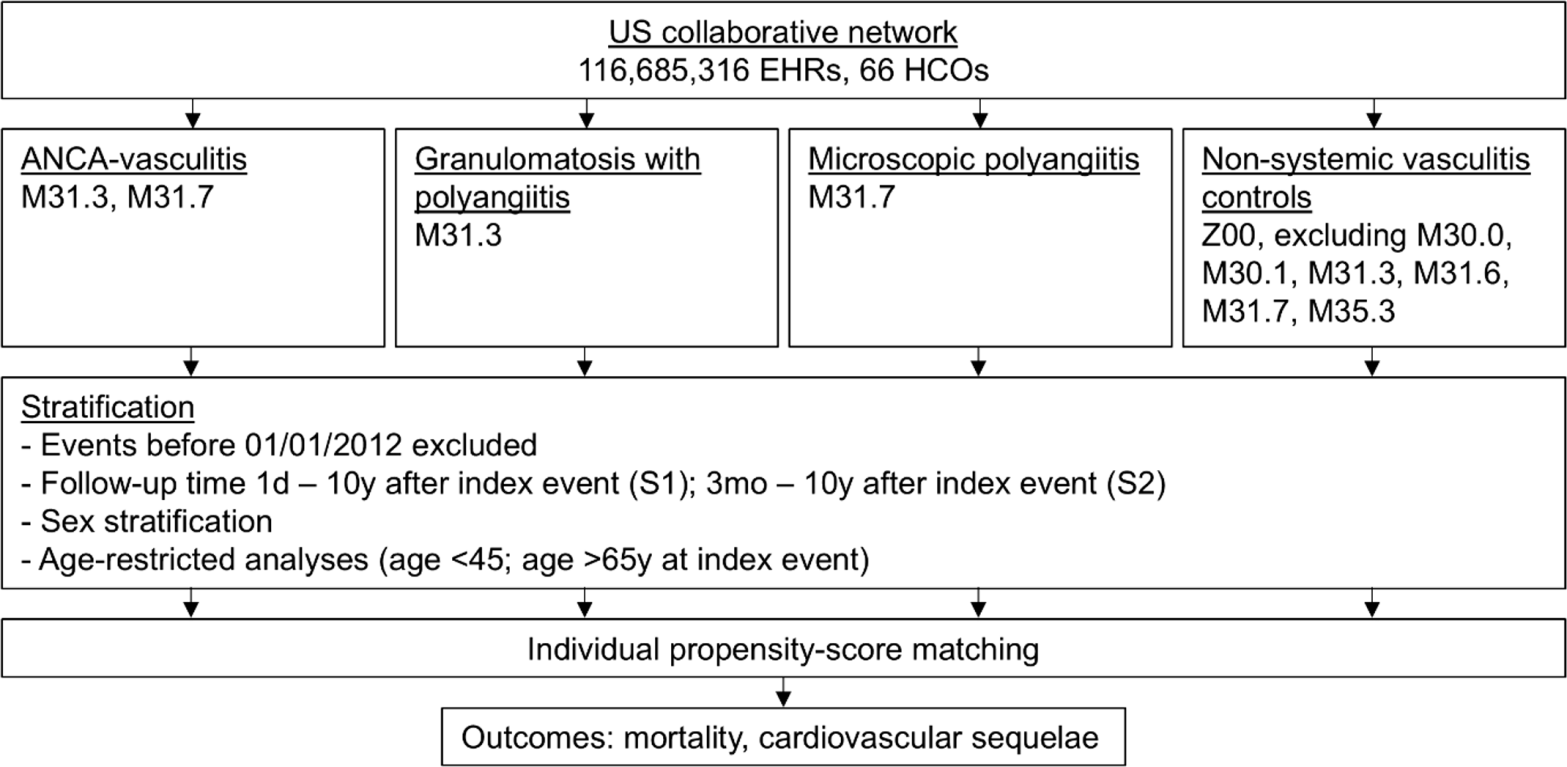
Flowchart study. EHRs, electronic health records; HCOs, Health Care Organizations.

The primary analysis compared the risk of death or cardiovascular diseases among the different cohorts at any time after the index event. In sensitivity analysis S1, EHRs between January 1^st^, 2012, and July 1^st^, 2024, were considered, when rituximab became increasingly preferred for induction and maintenance therapy in AAV, as is now the case (17). In S1, outcomes ranging from one day to 10 years after the index event were analyzed. Sensitivity analysis S2 was similar to S1, but excluded outcomes occurring within the first three years after the index event. To account for possible gender differences, sex-specific analyses were performed similarly to the primary analysis. In addition, age-restricted analyses were performed comparing EHRs of patients under 45 years of age with those over 65 years of age at the time of the index event, to address age-related cardiovascular outcomes defined by commonly used recommendations, i.e. the U.S. Preventive Services Task Force. The age-restricted analyses were also performed similarly to the primary analysis. All data were retrieved and analyzed in July 2024. The study design is depicted in **Figure 1**.

### Covariates

PSM was performed to control for relevant confounding variables. PSM is a well-established method to covariates between groups (18). We selected clinically relevant factors, including demographic information, cardiovascular risk factors, and other major diseases. Matching was performed 1:1 by the nearest neighbour greedy matching algorithm with a calliper distance of 0.1 standard deviations after establishing a propensity score for each EHR by logistic regression. Covariates included in the regression matrix as well as baseline characteristics before and after matching are presented in **Tables 1**–**3**.

**Table 1:** Baseline characteristics of patients with granulomatosis with polyangiitis and matched controls without vasculitis before and after propensity score matching.

Characteristic	Before matching				After matching			
	Cases (GPA)	Controls	P value	SMD	Cases (GPA)	Controls	*P* value	SMD
Number of participants	21, 190	7, 717, 202	–	–	20, 422	20, 422	n.s.	–
Age at Index (years, SD)	54.7 ± 18.3	34.4 ± 26.1	< 0.0001	0.902	54.7 ± 18.3	55.1 ± 18.4	0.0517	0.019
Female (%)	55.812	52.477	< 0.0001	0.067	55.812	55.592	0.6540	0.004
Family history of ischemic heart disease and other diseases of the circulatory system (%)	8.138	5.6	< 0.0001	0.100	8.138	7.702	0.1030	0.016
Personal history of nicotine dependence (%)	13.828	7.057	< 0.0001	0.223	13.828	13.441	0.2547	0.011
Nicotine dependence (%)	12.898	7.274	< 0.0001	0.188	12.898	12.305	0.0712	0.018
Overweight and obesity (%)	14.249	14.74	0.0481	0.014	14.249	13.867	0.2668	0.011
Disorders of lipoprotein metabolism and other lipidemias (%)	23.612	26.666	< 0.0001	0.070	23.612	23.764	0.7183	0.004
Essential (primary) hypertension (%)	31.696	24.467	< 0.0001	0.161	31.696	31.138	0.2243	0.012
Chronic lower respiratory diseases (%)	23.504	15.968	< 0.0001	0.233	23.504	22.931	0.1703	0.014
Chronic kidney disease (%)	22.662	4.31	< 0.0001	0.558	22.662	21.467	0.0036	0.029
Neoplasms (%)	26.055	19.706	< 0.0001	0.152	26.055	24.836	0.0047	0.028
Diabetes mellitus (%)	2.615	1.334	< 0.0001	0.092	2.615	2.84	0.1623	0.014

Baseline characteristics of the GPA cohort and the non-vasculitis control cohort before and after 1:1 propensity score matching. Categorical variables are presented as absolute and relative frequencies, and continuous variables as mean ± standard deviation (SD). P values compare cases and controls within the unmatched and matched cohorts, respectively.

GPA, granulomatosis with polyangiitis; SD, standard deviation; SMD, standardized mean differences.

**Table 2:** Baseline characteristics of patients with microscopic polyangiitis and matched controls without vasculitis before and after propensity score matching.

Characteristic	Before matching				After matching			
	Cases (MPA)	Controls	*P* value	SMD	Cases (MPA)	Controls	*P* value	SMD
Number of participants	5, 907	7, 717, 222	-	–	5, 907	5, 907	–	–
Age at Index (years, SD)	56.6 ± 20	34.4 ± 26.1	< 0.0001	0.953	56.6 ± 20	57.6 ± 19.5	0.0041	0.043
Female (%)	58.879	52.477	< 0.0001	0.129	58.879	59.15	0.7647	0.005
Family history of ischemic heart disease and other diseases of the circulatory system (%)	20.62	5.6	< 0.0001	0.456	20.62	20.603	0.9819	0.004
Personal history of nicotine dependence (%)	29.863	7.057	< 0.0001	0.615	29.863	29.677	0.8248	0.003
Nicotine dependence (%)	26.934	7.274	< 0.0001	0.541	26.934	26.917	0.9835	0.004
Overweight and obesity (%)	28.813	14.74	< 0.0001	0.346	28.813	28.932	0.8870	0.017
Disorders of lipoprotein metabolism and other lipidemias (%)	42.069	26.666	< 0.0001	0.329	42.069	43.068	0.2723	0.009
Essential (primary) hypertension (%)	58.084	24.467	< 0.0001	0.726	58.084	57.694	0.6682	0.013
Chronic lower respiratory diseases (%)	37.972	15.968	< 0.0001	0.512	37.972	37.43	0.5435	0.004
Chronic kidney disease (%)	57.017	4.31	< 0.0001	1.393	57.017	56.712	0.7381	0.005
Neoplasms (%)	43.508	19.706	< 0.0001	0.530	43.508	43.254	0.7807	0.025
Diabetes mellitus (%)	6.247	1.334	< 0.0001	0.682	6.247	6.298	0.9094	0.017

Baseline characteristics of the MPA and GPA cohorts before and after 1:1 propensity score matching for direct between-disease comparison. Categorical variables are presented as absolute and relative frequencies, and continuous variables as mean ± standard deviation (SD). P values compare the two disease cohorts within the unmatched and matched samples, respectively.

MPA, microscopic polyangiitis; GPA, granulomatosis with polyangiitis; SD, standard deviation; SMD, standardized mean differences.

**Table 3:** Baseline characteristics of patients with microscopic polyangiitis and granulomatosis with polyangiitis before and after propensity score matching.

Characteristic	Before matching				After matching			
	Cases (MPA)	Controls (GPA)	*P* value	SMD	Cases (MPA)	Controls (GPA)	*P* value	SMD
Number of participants	5, 907	21, 191	–	–	5, 907	5, 907	–	–
Age at Index (years, SD)	56.6 ± 20	54.7 ± 18.3	< 0.0001	0.079	56.6 ± 20	56.7 ± 19.3	0.6865	0.006
Female (%)	58.879	55.812	< 0.0001	0.063	58.879	58.608	0.7649	0.016
Family history of ischemic heart disease and other diseases of the circulatory system (%)	20.62	8.138	< 0.0001	0.354	20.62	20.163	0.5375	0.011
Personal history of nicotine dependence (%)	29.863	13.828	< 0.0001	0.390	29.863	29.609	0.7627	0.003
Nicotine dependence (%)	26.934	12.898	< 0.0001	0.353	26.934	27.425	0.5487	0.017
Overweight and obesity (%)	28.813	14.249	< 0.0001	0.352	28.813	28.627	0.8230	0.013
Disorders of lipoprotein metabolism and other lipidemias (%)	42.069	23.612	< 0.0001	0.387	42.069	41.984	0.9257	0.003
Essential (primary) hypertension (%)	58.084	31.701	< 0.0001	0.534	58.084	58.405	0.7230	0.006
Chronic lower respiratory diseases (%)	37.972	23.504	< 0.0001	0.311	37.972	37.904	0.9395	0.003
Chronic kidney disease (%)	57.017	22.662	< 0.0001	0.731	57.017	57.068	0.9555	0.001
Neoplasms (%)	43.508	26.055	< 0.0001	0.363	43.508	44.185	0.4583	0.005
Diabetes mellitus (%)	6.247	2.615	< 0.0001	0.181	6.247	5.976	0.5389	0.005

Baseline characteristics of the MPA and GPA cohorts before and after 1:1 propensity score matching for direct between-disease comparison. Categorical variables are presented as absolute and relative frequencies, and continuous variables as mean ± standard deviation (SD). P values compare the two disease cohorts within the unmatched and matched samples, respectively.

MPA, microscopic polyangiitis; GPA, granulomatosis with polyangiitis; SD, standard deviation; SMD, standardized mean differences.

### Model triangualtion

To provide additional adjustment beyond PSM and to support model triangulation, supplementary multivariable Cox proportional hazards models were performed for the two key endpoints, mortality and MACE, in GPA and MPA. Covariates included cohort membership (GPA or MPA), sex, age at index, family history of ischemic heart disease, smoking-related variables, overweight/obesity, disorders of lipoprotein metabolism, hypertension, diabetes mellitus, chronic lower respiratory disease, chronic kidney disease, and visit frequency as a proxy for health care utilization. Covariates were assessed before the index date, and outcomes were evaluated from 1 day after index onward.

### Statistical analysis

Follow-up time varied and as detailed in the description of the study design and database above. EHRs with an outcome prior to the index event were excluded from analysis. Baseline characteristics were summarized descriptively. Categorical variables are reported as absolute and relative frequencies (n, %), and continuous variables as mean ± standard deviation (SD). Relative risks and risk differences were calculated. Patients were censored after the last available record. Survival analyses were performed using the Kaplan-Meier (KM) method and Aalen-Johansen plots. The proportionality assumption was tested based on Schoenfeld residuals. KM-curves were compared by log-rank tests. Hazard ratios (HR) and confidence intervals (CI) were expressed by univariate Cox regressions. To adjust for multiple testing, the Bonferroni correction was used (α(adj)=0.003).

### Ethics statement

The data reviewed is a secondary analysis of existing data that does not involve any intervention or interaction with human subjects. The study was conducted and reported according to the STROBE guidelines (19).

## Results

Among 116, 685, 316 EHRs of the TriNetX database at baseline and over 3 million EHRs per group, 21, 190 patients with GPA and 5, 907 patients with MPA were identified as defined by the inclusion and exclusion criteria (**Figure 1**). The mean age at diagnosis was 54.7 years (SD 18.3) for the GPA group and 56.6 years (SD 20.0) for the MPA group. 55.8% of the GPA patients and 58.9% of the MPA patients were female. After propensity score matching for demographic variables and cardiovascular outcomes, the risk of mortality and cardiovascular events was assessed in 20, 422 GPA patients (**Table 1**) and 5, 907 MPA patients (**Table 2**) compared to matched controls.

**Figure 2:**
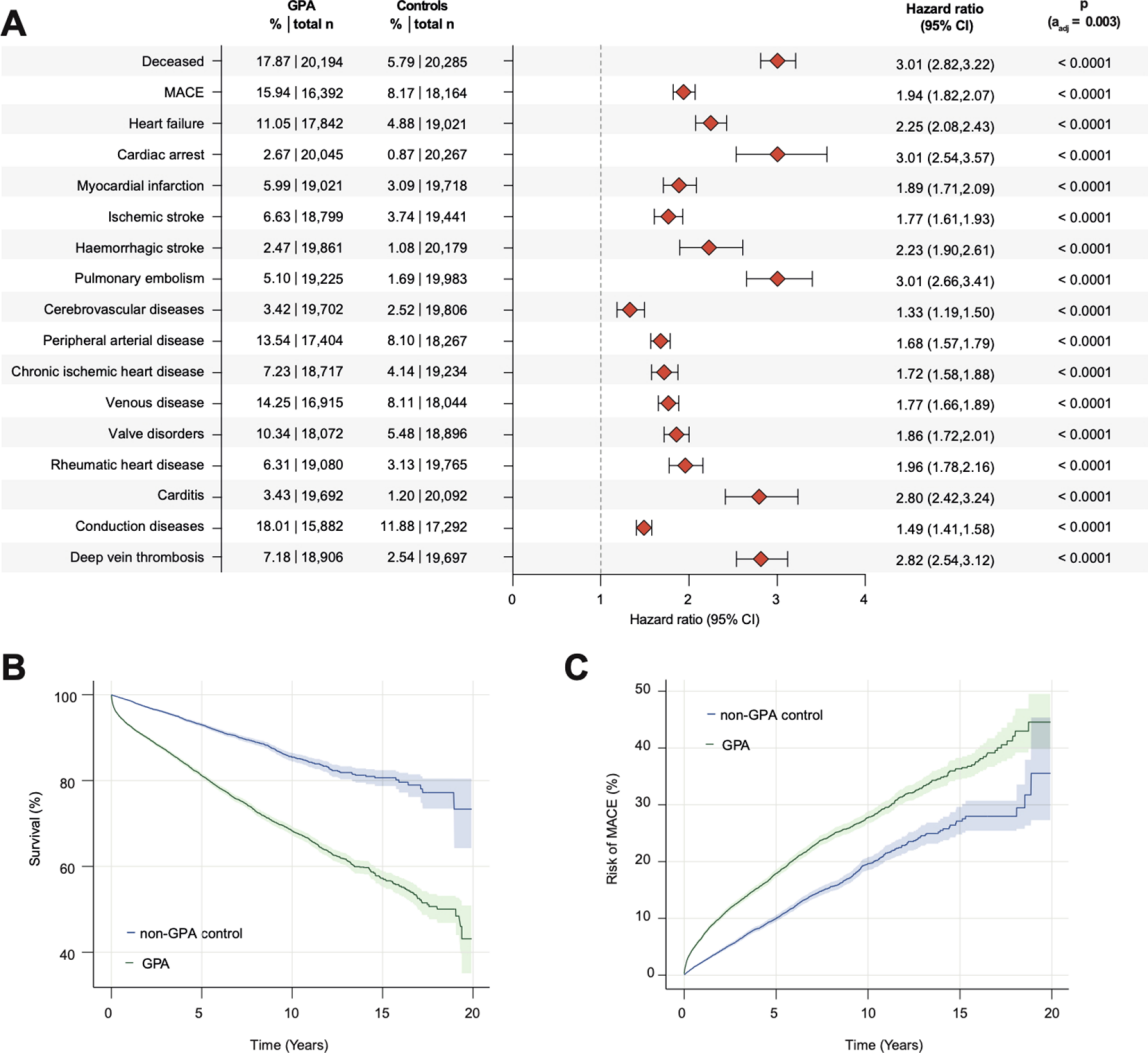
Risk of death and cardiovascular outcomes in GPA. **(A)** Forest plot depicting HRs for mortality and cardiovascular morbidity in GPA compared to matched controls (shading indicates 95%CI). Indicated p-value refers to log rank test results; **(B)** Cumulative probability of survival in GPA compared to matched controls; **(C)** Cumulative probability of MACE (acute cerebral and myocardial infarction, congestive heart failure, ventricular arrhythmia and sudden cardiac death) in GPA compared to matched controls. Abbreviations: GPA, granulomatosis with polyangiitis; MACE: major adverse cardiovascular events.

**Figure 3:**
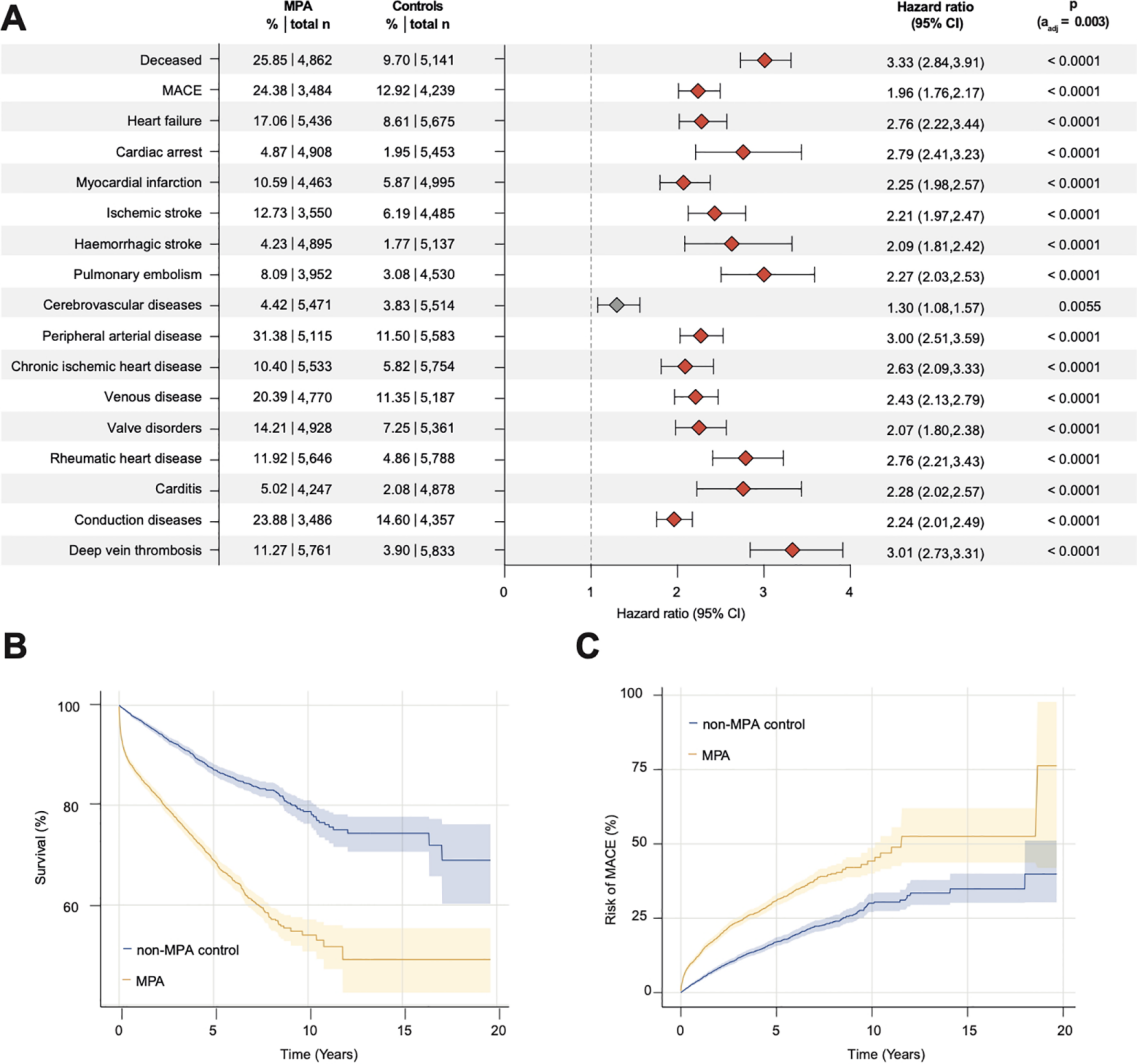
Risk of death and cardiovascular outcomes in MPA. **(A)** Forest plot depicting HRs for mortality and cardiovascular morbidity in GPA compared to matched controls (shading indicates 95%CI); **(B)** Cumulative probability of survival in MPA compared to matched controls; **(C)** Cumulative probability of MACE (acute cerebral and myocardial infarction, congestive heart failure, ventricular arrhythmia and sudden cardiac death) in MPA compared to matched controls. Abbreviations: MPA, microscopic polyangiitis; MACE, major adverse cardiovascular events.

In multivariable models, the risk of mortality was increased in GPA (17.87%) and MPA (25.85%) compared with matched controls (GPA: 5.79%, HR 3.01, 95% CI 2.82–3.22, **Figure 2**; MPA 9.70%, HR 3.33, 2.84-3.91, **Figure 3**). To exclude therapeutic effects, particularly given the widespread availability of rituximab, electronic health records (EHRs) of GPA and MPA patients (n = 21, 195) from January 1, 2012, were used for a sensitivity analysis. Here, the risk of mortality remained increased compared to matched controls (HR, 3.01; 95% CI, 2.82–3.21; **Supplementary Table 1**). Regarding the first three years after diagnosis, a higher risk of mortality was found in GPA and MPA patients (n = 22, 720) compared with matched controls (HR: 3.52, 95% CI: 3.24–3.81; **Supplementary Table 2**). To address gender-specific risks, female (n = 12, 822) and male (n = 9, 457) GPA and MPA patients were analyzed separately. Compared to the matched controls, the risk of mortality was increased in both females and males (female GPA and MPA: HR 3.56, 95% CI 3.15–4.02, **Supplementary Table 3**; male GPA and MPA: HR 3.26, 95% CI 2.92–3.64, **Supplementary Table 4**). To account for possible age-related effects, GPA and MPA patients were segregated into two groups: those under 45 years of age (n = 4, 564) and those over 65 years of age (n = 4, 430). Both younger and elder patients had a higher risk for mortality compared to matched controls (GPA and MPA younger than 45 years: HR 5.24, 95% CI 3.50–7.85, **Supplementary Table 5**; GPA and MPA older than 65 years: HR 5.25, 95% CI 3.50-7.86, **Supplementary Table 6**). Thus, there was no significant difference in the risk of mortality between GPA and MPA patients (n = 5, 907, **Table 3**) after propensity score matching for demographic variables and cardiovascular comorbidity (HR 1.11, 95% CI 1.03-1.19, **Figure 3**).

**Figure 4:**
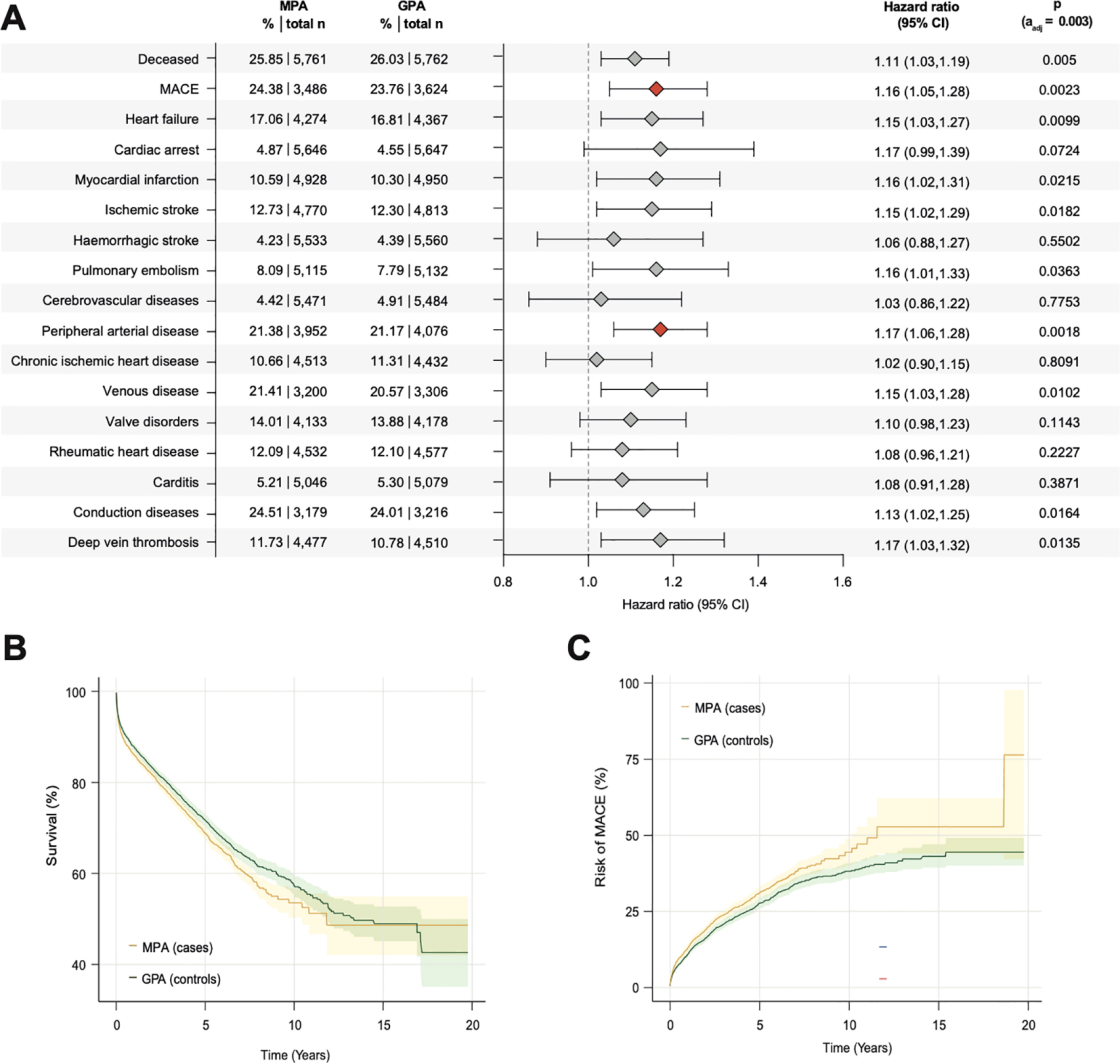
Risk of death and cardiovascular outcomes in MPA compared to GPA. **(A)** Forest plot depicting HRs for mortality and cardiovascular morbidity in patients with MPA compared to matched patients with GPA (shading indicates 95%CI); **(B)** Cumulative probability of survival in MPA compared to GPA; **(C)** Cumulative probability of MACE (acute cerebral and myocardial infarction, congestive heart failure, ventricular arrhythmia and sudden cardiac death) in MPA compared to GPA. Abbreviations: MPA, microscopic polyangiitis; GPA, granulomatosis with polyangiitis; MACE, major adverse cardiovascular events.

All screened cardiovascular comorbidities were increased in GPA and MPA patients, regardless of gender, age, or time since diagnosis (**Figure 2** and **3**; **Supplementary Tables 1**–**6**). In particular, the risk of cardiac arrest, pulmonary embolism, carditis and deep vein thrombosis was significantly increased compared to matched controls. This was especially evident in patients under 45 years of age (cardiac arrest: HR 10.92, 95% CI 3.35-35.59; pulmonary embolism: HR 9.12, 95% CI 5.02-16.57; carditis: HR 4.89, 95% CI 2.94-8.13; deep vein thrombosis: HR 7.68, 95% CI 4.87-12.11; **Supplementary Table 5**). In addition, an increased risk of major adverse cardiovascular events (MACE; acute cerebral and myocardial infarction, congestive heart failure, ventricular arrhythmia and sudden cardiac death) and peripheral arterial disease was observed in patients with MPA compared to matched GPA patients (MACE: HR 1.16, 95% CI 1.05-1.28; peripheral arterial disease: HR 1.17, 95% CI 1.06-1.28, **Figure 4**). For model triangulation, supplementary multivariable Cox proportional hazards analyses were performed and showed that MPA remained associated with higher hazards of mortality (HR 1.23, 95% CI 1.13–1.33; p<0.0001) and MACE (HR 1.37, 95% CI 1.29–1.47; p<0.0001) compared with GPA (**Supplementary Table 7**). Although not statistically significant, MPA patients showed a trend towards an increased risk of thromboembolic events (pulmonary embolism: HR 1.16, 95% CI 1.01 -1.33; deep vein thrombosis: HR 1.17, 95% CI 1.03-1.32) and the risk of heart failure compared to GPA (HR 1.15, 95% 1.03-1.27) (**Figure 3**).

## Discussion

Our findings show that, compared to individuals matched by propensity score, patients with GPA or MPA had an increased mortality and cardiovascular disease risk. Even in patients under 45 years of age, the risk of cardiac arrest, pulmonary embolism, carditis, and deep vein thrombosis was increased. Furthermore, patients with MPA were found to be at an increased risk for major adverse cardiac events (MACE) and peripheral disease compared to patients with GPA. These findings suggest a disease-specific comorbidity in AAV.

Previous studies showed increased mortality due to active disease, infections, and cardiovascular events in AAV (4–9). In particular, long-term follow-up data from 848 AAV patients from seven different randomized controlled clinical trials conducted between 1995 and 2012 revealed increased mortality in all age groups caused by infections, malignancies, and cardiovascular events (4). Negative prognostic factors included male sex and age over 65 years. Our study confirmed an increased mortality risk in AAV patients, but did not show a significantly increased risk in male patients as compared with female patients. To account for possible age-related effects, we compared the group of GPA and MPA patients under 45 years of age with those over 65 years of age. In the present study, we found an increased risk of death in patients both under 45 and over 65 years of age, underscoring a need for cardiovascular monitoring for both younger and elder AAV patients. Therefore, younger patients with AAV, in particular, should be screened for other cardiovascular risks, such as arterial hypertension. Close monitoring should also be carried out throughout the course of the disease with regard to other cardiovascular risk factors. Therefore, prospective controlled studies should also evaluate whether AAV itself might be a cardiac risk factor. A previous study had also observed an increased risk of death in MPA compared to GPA in a monocentric retrospective analysis of 254 AAV patients diagnosed between 1997 and 2021 (6). Our study based on propensity score matching for demographic variables and comorbidity did not confirm those findings. However, patients with MPA were at higher risk for MACE and peripheral arterial disease compared with GPA patients in the present study.

Persistent systemic inflammation and autoimmune pathways are well-known risk factors for cardiovascular disease and are associated with increased mortality (20). Various multicenter and retrospective meta-analyses have described cardiovascular comorbidities in AAV patients. In particular, AAV patients had an increased risk of ischemic heart disease (7, 10, 21, 22), heart failure (8–11, 21), cerebrovascular disease (7, 22), myocardial infarction (8, 10, 21, 23), cardiac arrest (10), deep vein thrombosis (10), pulmonary embolism (10), ischemic or hemorrhagic stroke (9, 10, 21, 22, 24), MACE (21), venous thromboembolism (21), pericarditis/carditis (21) and peripheral arterial disease (25). However, the available data were limited, as was the width of cardiovascular morbidity and the quality of matching. Furthermore, the cardiovascular risks were not uniformly increased in the cohorts studied. In detail, a nationwide cohort study of 1, 923 Danish GPA patients identified an increased risk of screened cardiovascular outcomes, which was limited to the first year after diagnosis (9). Furthermore, a population-based cohort study of 1, 520 Canadian AAV patients confirmed an increased risk of developing atrial fibrillation and stroke, but not MACE or myocardial infarction (11). This creates an inconsistent situation due to the use of heterogeneous data sources. In contrast, the present study overcame this limitation by using a large global database comprising a wide range of analyzed cardiovascular comorbidities. The present study is an expansion of extant knowledge regarding the elevated risk of cardiovascular disease in AAV, achieved by means of an exhaustive examination of a substantial number of comorbidities and an enlarged patient sample. This comprehensive approach facilitates analysis across diverse age demographics.

To date, no studies have examined the impact of cardiovascular events on patients with MPA or GPA. This study is the first to provide evidence that MPA patients are at an increased risk of major adverse cardiac events (MACE) and peripheral arterial disease compared to GPA patients. This finding is based on an expanded cohort of over 5, 100 patients, matched using propensity scores for demographic variables and comorbidities. It is important to note that, in contrast to the findings reported in (26), the present study compared GPA and MPA patients of almost the same age, thereby eliminating the effect of age difference. The elevated risk of MACE and peripheral arterial disease may be associated with arterial stiffness (27), which could be a leading cause of cardiovascular death in AAV due to T-cell-mediated, autoimmunity-associated cardiovascular disease (28). As a clinically relevant extension to previous studies, AAV patients should also be examined for MACE and peripheral arterial disease using specific examination techniques (28). Therefore, future recommendations should be reviewed to determine whether extended screening for MACE and peripheral arterial disease, especially in MPA, should be included at defined time points, regardless of the presence of clinical symptoms, in order to address subclinical manifestations.

Our study has several strengths. It comprises a large sample size with over 3 million EHRs per group. This extensive dataset allowed for detailed PSM to mitigate bias from potential confounders. In contrast to multicenter studies and retrospective meta-analyses, the present study provides real-world evidence on cardiovascular risk factors and risk of mortality in large cohort of patients. Limitations such as the retrospective design, which precludes causal inference from the reported associations, must also be considered. Despite extensive cohort matching, both measured and unmeasured confounders may still distort the results. Although statistical significance levels were adjusted for multiplicity, competing risks, especially when analyzing multiple cardiovascular outcomes, cannot be fully controlled due to the aggregated nature of the underlying data. Another possible confounder may arise from miscoding or non-coding of diagnoses, particularly given the changes in diagnostic and classification criteria over this large time span. Furthermore, when using EHRs, limitations regarding the influence of specific organ involvement and serological findings, particularly the ANCA profile, must be considered. Therapeutic interventions are critical for outcomes such as disease activity and therapy-associated outcomes (4–7). In the present study, we excluded subgroup analyses regarding the type of remission induction and maintenance therapy, as well as the glucocorticoid dosage, due to the heterogeneity of coded medications and the potential risk of missing information. To minimise the potential influence of heterogeneous healthcare systems, our study focused on comparable data from HCOs in Europe and the US.

Patients with eosinophilic granulomatosis with polyangiitis (EGPA), the rarest AAV, were excluded from this study due to the heterogeneity of the condition. Consequently, our study focused on mortality and cardiovascular risk related to GPA and MPA, respectively. Furthermore, psychosocial and geographic factors may influence the level of engagement with healthcare providers, potentially leading to the underrepresentation of certain population, segments in the data.

In conclusion, our study demonstrated an increase in mortality and cardiovascular morbidity in AAV patients, regardless of duration, gender, or age. Our findings emphasize the high-risk nature of this population concerning adverse cardiovascular outcomes. MPA was found to be associated with a higher risk of MACE and peripheral arterial disease compared to GPA highlighting disease-specific cardiovascular risk profiles in the two AAV subgroups. Further analysis using prospective observational cohorts is required to corroborate these findings.

## Data Availability

The original contributions presented in the study are included in the article/**Supplementary Material**. Further inquiries can be directed to the corresponding author.
