# Supplementary material for: ANCA-associated vasculitis is associated with an increased risk of cardiac and vascular morbidity: results of a large-scale propensity-matched global retrospective cohort study
Source: Front Immunol. 2026 Apr 15;17:1794549. doi: 10.3389/fimmu.2026.1794549 (PMC13124934; doi:10.3389/fimmu.2026.1794549)
Supplement: Supplementary file 4 [file Table4.docx]

| **Outcome** | **Cases**  **(GPA or MPA)** | | | **Controls** | | | **Analysis** | | | | |
| --- | --- | --- | --- | --- | --- | --- | --- | --- | --- | --- | --- |
|  | **N of eligible participants** | **N of Out-comes** | **Risk, %** | **N of eligible participants** | **N of Out-comes** | **Risk, %** | **Risk difference, %** | **(95% confidence interval)** | **Hazard ratio** | **(95% confidence interval)** | **P value**  **(α_adj._= 0.003)** |
| Deceased | 9,329 | 1,299 | 13.924 | 9,387 | 427 | 4.549 | 9.375 | (8.556,10.195) | 3.264 | (2.926,3.642) | < 0.0001 |
| MACE | 7,234 | 855 | 11.819 | 8,185 | 427 | 5.217 | 6.602 | (5.716,7.489) | 2.44 | (2.172,2.74) | < 0.0001 |
| Heart failure | 8,012 | 663 | 8.275 | 8,637 | 269 | 3.115 | 5.161 | (4.455,5.866) | 2.848 | (2.472,3.282) | < 0.0001 |
| Cardiac arrest | 9,212 | 199 | 2.16 | 9,355 | 67 | 0.716 | 1.444 | (1.101,1.787) | 3.187 | (2.416,4.203) | < 0.0001 |
| Myocardial infarction | 8,625 | 390 | 4.522 | 9,012 | 179 | 1.986 | 2.535 | (2.011,3.06) | 2.434 | (2.039,2.906) | < 0.0001 |
| Ischemic stroke | 8,671 | 410 | 4.728 | 8,953 | 189 | 2.111 | 2.617 | (2.08,3.154) | 2.408 | (2.027,2.861) | < 0.0001 |
| Haemorrhagic stroke | 9,162 | 170 | 1.855 | 9,324 | 67 | 0.719 | 1.137 | (0.812,1.462) | 2.754 | (2.075,3.653) | < 0.0001 |
| Pulmonary embolism | 8,874 | 332 | 3.741 | 9,226 | 97 | 1.051 | 2.69 | (2.244,3.136) | 3.815 | (3.043,4.784) | < 0.0001 |
| Cerebrovascular diseases | 9,077 | 233 | 2.567 | 9,125 | 150 | 1.644 | 0.923 | (0.506,1.34) | 1.674 | (1.364,2.056) | < 0.0001 |
| Peripheral arterial disease | 7,932 | 738 | 9.304 | 8,282 | 425 | 5.132 | 4.172 | (3.376,4.969) | 1.981 | (1.758,2.232) | < 0.0001 |
| Chronic ischemic heart disease | 8,447 | 478 | 5.659 | 8,747 | 264 | 3.018 | 2.641 | (2.031,3.25) | 2.006 | (1.726,2.332) | < 0.0001 |
| Venous disease | 7,732 | 781 | 10.101 | 8,397 | 391 | 4.656 | 5.444 | (4.636,6.253) | 2.361 | (2.091,2.666) | < 0.0001 |
| Valve disorders | 8,222 | 615 | 7.48 | 8,724 | 301 | 3.45 | 4.03 | (3.344,4.715) | 2.326 | (2.027,2.67) | < 0.0001 |
| Rheumatic heart disease | 8,783 | 375 | 4.27 | 9,121 | 146 | 1.601 | 2.669 | (2.174,3.164) | 2.831 | (2.339,3.428) | < 0.0001 |
| Carditis | 9,078 | 248 | 2.732 | 9,274 | 71 | 0.766 | 1.966 | (1.587,2.346) | 3.796 | (2.916,4.943) | < 0.0001 |
| Conduction diseases | 6,989 | 911 | 13.035 | 7,715 | 553 | 7.168 | 5.867 | (4.89,6.844) | 1.942 | (1.748,2.159) | < 0.0001 |
| Deep vein thrombosis | 8,671 | 461 | 5.317 | 9,125 | 118 | 1.293 | 4.023 | (3.497,4.55) | 4.411 | (3.603,5.4) | < 0.0001 |

**Supplement Table 4**. Risk of death, and cardiovascular outcomes in cases (diagnosis of granulomatosis with polyangiitis [GPA] or microscopic polyangiitis [MPA]) and controls for subgroup analysis considering only male patients. Electronic health records (EHRs) of cases and controls were retrieved from the US Collaborative Network of TriNetX. Propensity score matching for demographic variables and comorbidity was performed to optimize between-group comparability. Hazard ratios were calculated by univariate Cox regression after analysis of the matched cohort by the Kaplan-Meier method. P values were determined by the Log-rank test. Data was retrieved on July 2024.

portional hazards model
