# Supplementary material for: ANCA-associated vasculitis is associated with an increased risk of cardiac and vascular morbidity: results of a large-scale propensity-matched global retrospective cohort study
Source: Front Immunol. 2026 Apr 15;17:1794549. doi: 10.3389/fimmu.2026.1794549 (PMC13124934; doi:10.3389/fimmu.2026.1794549)
Supplement: Supplementary file 7 [file Table7.docx]

| Multivariable Cox proportional hazards model |  |  |  |  |
| --- | --- | --- | --- | --- |
| Outcome: Mortality |  |  |  |  |
| Sample size - MPA: 4,101 |  |  |  |  |
| Sample size - CoGPA: 19,308 |  |  |  |  |
| Model: Multivariable Cox proportional hazards regression |  |  |  |  |
| Adjustment variables: Age at index; sex; family history of ischemic heart disease and other diseases of the circulatory system; personal history of nicotine dependence; nicotine dependence; overweight and obesity; disorders of lipoprotein metabolism and other lipidemias; essential hypertension; diabetes mellitus; chronic lower respiratory diseases; chronic kidney disease; visit | | | | |
|  |  |  |  |  |
| **Covariate** | **Hazard Ratio** | **Lower 95% CI** | **Upper 95% CI** | **P value** |
| MPA (cases) versus GPA (controls) | 1,229 | 1,133 | 1,333 | <0.0001 |
| Male | 1,301 | 1,22 | 1,388 | <0.0001 |
| Age at index | 1,052 | 1,049 | 1,055 | <0.0001 |
| Family history of ischemic heart disease and other diseases of the circulatory system | 1,184 | 1,026 | 1,366 | 0,0207 |
| Personal history of nicotine dependence | 1,086 | 0,986 | 1,195 | 0,0928 |
| Nicotine dependence | 1,409 | 1,259 | 1,578 | <0.0001 |
| Overweight and obesity | 0,953 | 0,857 | 1,06 | 0,378 |
| Disorders of lipoprotein metabolism and other lipidemias | 0,884 | 0,812 | 0,964 | 0,005 |
| Essential (primary) hypertension | 1,011 | 0,927 | 1,101 | 0,8104 |
| Diabetes mellitus | 1,278 | 1,168 | 1,397 | <0.0001 |
| Chronic lower respiratory diseases | 1,198 | 1,106 | 1,297 | <0.0001 |
| Chronic kidney disease (CKD) | 1,671 | 1,542 | 1,809 | <0.0001 |
| Visit | 0,788 | 0,71 | 0,875 | <0.0001 |

**Supplement Table 7** Multivariable Cox proportional hazards model
